# DAPL1 is a novel regulator of testosterone production in Leydig cells of mouse testis

**DOI:** 10.1038/s41598-021-97961-6

**Published:** 2021-09-17

**Authors:** Hong-bin Chen, Jorge Carlos Pineda Garcia, Shinako Arizono, Tomoki Takeda, Ren-shi Li, Yukiko Hattori, Hiroe Sano, Yuu Miyauchi, Yuko Hirota, Yoshitaka Tanaka, Yuji Ishii

**Affiliations:** 1 Laboratory of Molecular Life Sciences, Graduate School of Pharmaceutical Sciences, Kyushu University, 3-1-1 Maidashi, Higashi-ku, Fukuoka, 812-8582 Japan; 2 Division of Pharmaceutical Cell Biology, Graduate School of Pharmaceutical Sciences, Kyushu University, Fukuoka, Japan; 3 Sino-Jan Joint Lab of Natural Health Products Research, School of Traditional Chinese Medicines, China Pharmaceutical University, Nanjing, China; 4 Division of Experimental, Japan Bioassay Research Center, Japan Organization of Occupational Health and Safety, Hadano, Japan

**Keywords:** Steroid hormones, Gonadal hormones

## Abstract

Leydig cells in the testes produce testosterone in the presence of gonadotropins. Therefore, male testosterone levels must oscillate within a healthy spectrum, given that elevated testosterone levels augment the risk of cardiovascular disorders. We observed that the expression of death-associated protein-like 1 (DAPL1), which is involved in the early stages of epithelial differentiation and apoptosis, is considerably higher in the testes of sexually mature mice than in other tissues. Accordingly, *Dapl1*-null mice were constructed to evaluate this variation. Notably, in these mice, the testicular levels of steroidogenic acute regulatory protein (StAR) and serum testosterone levels were significantly elevated on postnatal day 49. The findings were confirmed in vitro using I-10 mouse testis-derived tumor cells. The in vivo and in vitro data revealed the DAPL1-regulated the expression of StAR involving altered transcription of critical proteins in the protein kinase A and CREB/CREM pathways in Leydig cells. The collective findings implicate DAPL1 as an important factor for steroidogenesis regulation, and DAPL1 deregulation may be related to high endogenous levels of testosterone.

## Introduction

In male mammals, approximately 95% of the body’s testosterone is produced by Leydig cells located within interstitial compartments of the testis^1^. Endocrine control of this process is exerted via the hypothalamus–pituitary–gonadal (HPG) axis, in which gonadotropin-releasing hormone (GnRH) from the hypothalamus triggers the secretion of follicle-stimulating hormone (FSH) and luteinizing hormone (LH) from the pituitary. LH then binds to the LH receptor (LHR) in Leydig cells to promote steroid synthesis. Simultaneously, testosterone inhibits the secretion of LH and GnRH through a negative feedback loop. Therefore, it is crucial that male testosterone levels are preserved within a healthy range. In terms of testosterone levels, numerous studies have investigated insufficient testosterone production in males. However, the literature regarding excessive testosterone production remains scarce, with limited studies focusing on elevated testosterone levels. It should be noted that although a correlation between high testosterone levels and prostate cancer has been explored, the findings remain controversial^2–4^. Moreover, a recent study has highlighted that high testosterone levels could be linked to a higher risk for cardiovascular events in male subjects^5^.

Steroidogenesis is a multi-enzyme complex process through which precursor cholesterol is transformed into biologically active steroid hormones in a tissue-specific manner. The binding of LH to the LHR on Leydig cells stimulates Gs protein and activates adenylate cyclase, thereby increasing cyclic adenosine monophosphate (cAMP) levels. Cyclic AMP acts as a key second messenger and upregulates the expression of genes related to steroid production through the protein kinase A (PKA) pathway^6^. Importantly, cAMP-response element-binding protein (CREB) and CRE modulator protein (CREM) increase steroidogenic acute regulatory protein (StAR) expression^7^. StAR is a member of the START domain protein family, which mediates cholesterol transport from the outer to the inner mitochondrial membrane^8^. StAR-mediated cholesterol transport is a key step in steroid formation^9,10^, and a precise cAMP concentration is necessary to regulate StAR expression^11^. In Leydig cells, in addition to the cAMP/PKA pathway, which regulates StAR expression, other factors, including steroidogenic factors, the protein kinase C pathway, and the mitogen-activated protein kinase (MAPK)/extracellular-signal-regulated kinase (ERK) pathway, are also associated with StAR regulation^12^.

Death-associated protein-like 1 (DAPL1) was first found to be expressed in cells immediately above the proliferative compartment in various epithelia such as the corneal epithelium, epidermis, and tongue epithelium, and it is speculated that it plays a role during the early stages of epithelial cell differentiation^13^. It has recently been reported that DAPL1 can inhibit the proliferation of retinal pigment epithelium cells by regulating cell cycle-related proteins^14,15^. Nevertheless, little is known regarding the physiological functions of DAPL1 in other tissues presenting high expression. The initial experiments revealed that, in sexually mature mice, *Dapl1* expression was higher in the testis than in other tissues. In the present study, *Dapl1*-knockout (KO) mice were constructed using the Crisper-Cas9 method^16^. After confirming the successful establishment of desired deletions, *Dapl1*-KO mice were characterized by comparing with wild-type C57BL/6 J mice.

## Results

### DAPL1 is highly expressed in the pituitary gland-gonadal axis, and expression may present gender differences

**Figure 1:**
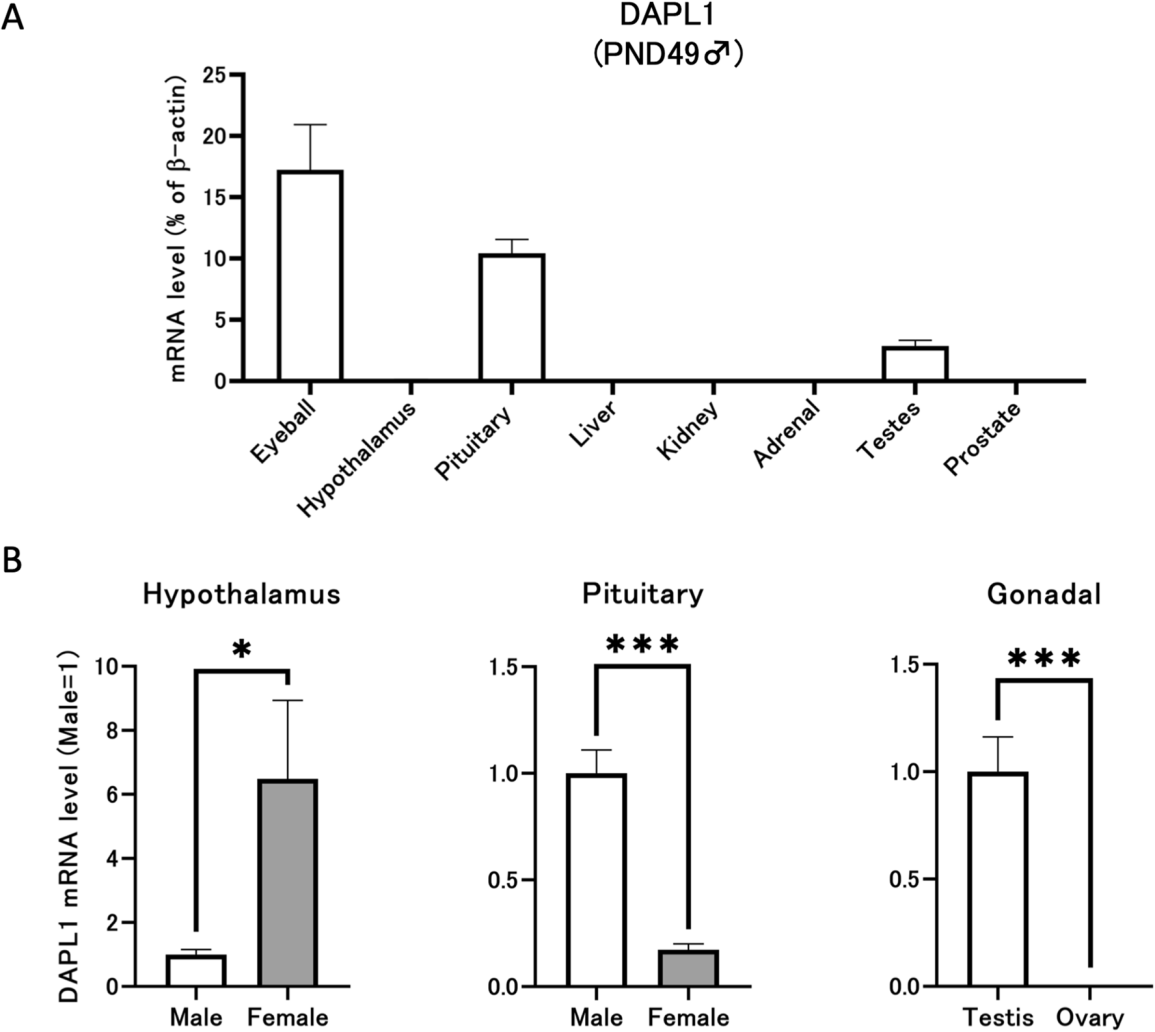
Comparison of DAPL1 mRNA expression levels between different tissues and in the same tissue from different genders. (**A**) DAPL1 mRNA expression levels in different tissues from adult mice were compared. Each bar represents the mean ± S.E.M. of 3–9 mice. (**B**) The mRNA expression of DAPL1 in the hypothalamus–pituitary–gonadal axis of adult wild-type mice were compared between male and female. Each bar represents the mean ± S.E.M. of 5–6 mice. Significantly different from the male group: *p < 0.05, ***p < 0.001. *DAPL1* death-associated protein-like 1.

**Figure 2:**
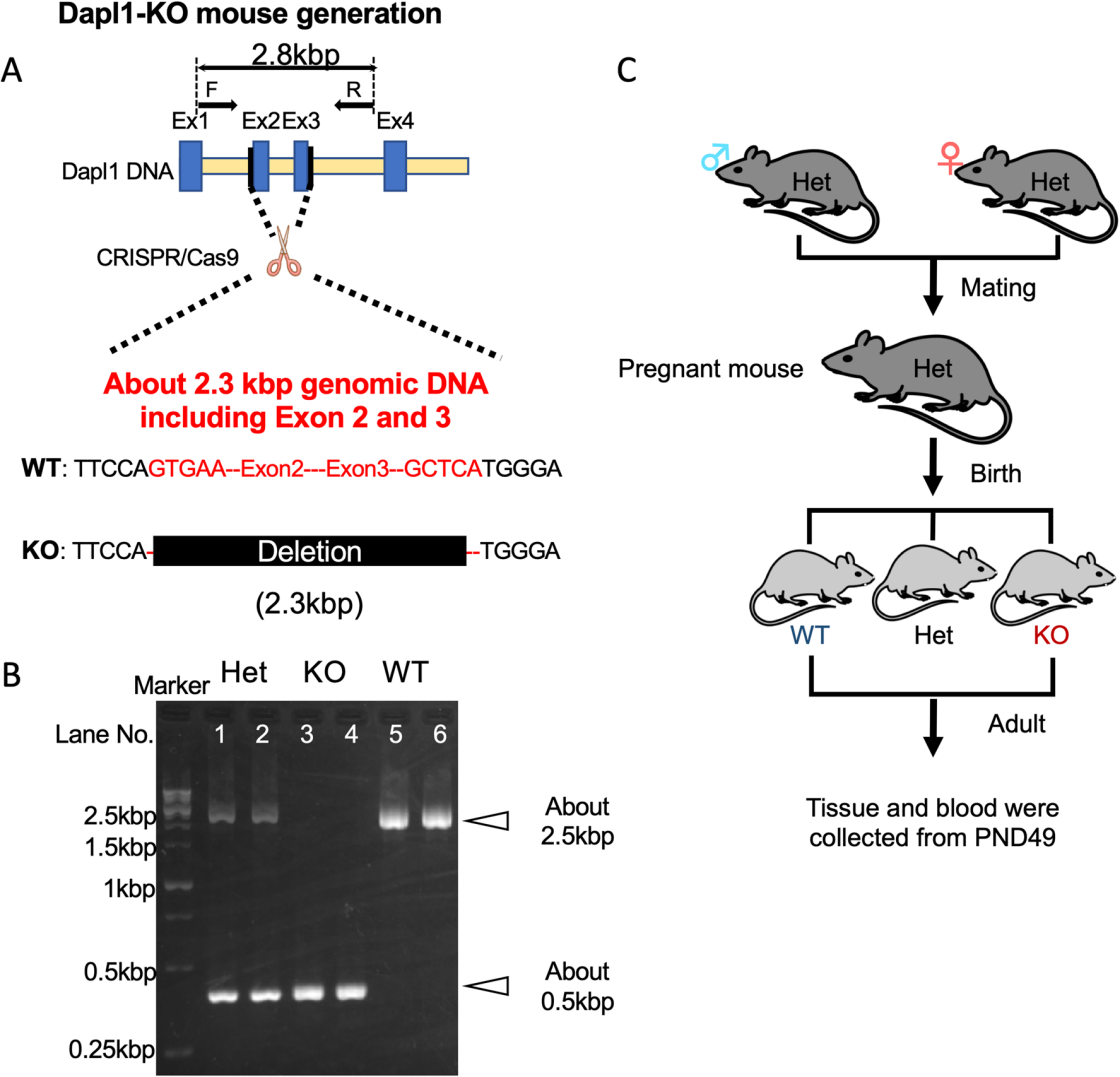
Experimental design and confirmation of *Dapl1* deletion. (**A**) Schematic representation for the CRISPER/Cas9-mediated gene deletion used for the generation of *Dapl1*-KO mice. F and R corresponds to *Dapl1* Forward and Reverse primers respectively, for the coding region of interest employed for assessing genotyping experiments. (**B**) The diagnostic patterns of three *Dapl1* genotypes are shown. Full-length agarose gel for (**B**) is presented in Supplementary Fig. S6. (**C**) Experimental design of the study. *Ex* exon, *KO* homo-knockout, *Het* heterozygote, *WT* wild-type, *PND* postnatal day. Some illustrations used in this figure were originated from leased Motifolio (Scientific Illustration Toolkits for Presentations and Publications) materials.

We attempted to elucidate differences in DAPL1 expression levels in various tissues. Accordingly, specific tissues and organs derived from adult male mice were used to extract total RNA for detection by reverse transcription-polymerase chain reaction (RT-PCR). DAPL1 was highly expressed in the cornea, consistent with previous reports^13^. Simultaneously, mRNA expression levels of DAPL1 in the pituitary and testis were considerably higher than those of other tested tissues, except for the cornea (Fig. 1A). Therefore, It can be postulated that DAPL1 may be related to the HPG axis. Next, we compared the mRNA expression levels of DAPL1 in the HPG axis in wild-type adult male and female mice. The results revealed that the expression of DAPL1 in male gonads was much higher than that in females (Fig. 1B). Even though these results are limited to DAPL1 expression, it is plausible to hypothesize that DAPL1 may exhibit diverse influences on male testes. However, further research is required to elucidate the extent and specificity of these possible influences. Subsequently, to further asses the expression of DAPL1 we proceed to construct *Dapl1*-KO mices via CRISPER/Cas9-mediated gene deletion (Fig. 2).

### *Dapl1* deletion can increase StAR expression in mouse testes, thereby affecting steroid synthesis and increasing endogenous testosterone levels

**Figure 3:**
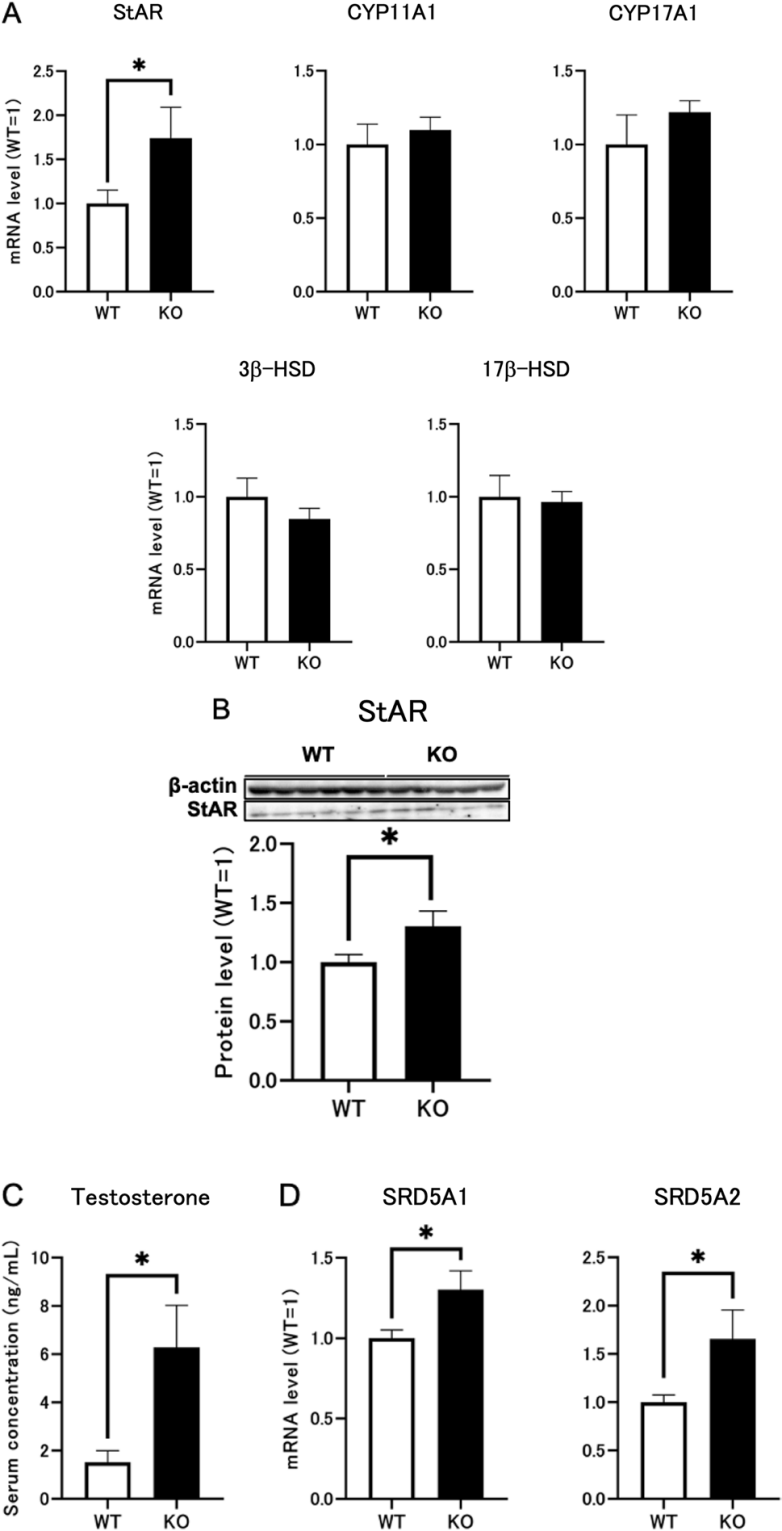
Effects of *Dapl1* deletion on sex steroid hormone synthesis in mouse testes at PND49. (**A**) Effect of *Dapl1* ablation on the testicular steroidogenesis gene-expression of mRNA coding for StAR, CYP11A1, CYP17A1, 3β-HSD, and 17β-HSD in male mice at PND49. Each bar represents the mean ± S.E.M. of 9–12 mice. (**B**) Effect of *Dapl1* ablation on the testicular expression of StAR protein in the mice at PND49. The representative electrophoretic image of each group is shown in the upper panels. The relative level of StAR expression was calculated by normalization to β-actin. Each bar represents the mean ± S.E.M. of 5–6 mice. (**C**) Effect of *Dapl1* ablation on circulating levels of testosterone in male mice at PND49. Each bar represents the mean ± S.E.M. of 6–7 mice. (**D**) Effect of *Dapl1* ablation on the testicular steroidogenesis gene-expression of mRNA coding for SRD5A1 and SRD5A2 in male mice at PND49. Each bar represents the mean ± S.E.M. of 9–11 mice. Significantly different from the wild-type control: *p < 0.05. *DAPL1* death-associated protein-like 1, *StAR* steroidogenic acute regulatory protein, *CYP* cytochrome P450, *3β-HSD* 3β-hydroxysteroid dehydrogenase, *17β-HSD* 17β-hydroxysteroid dehydrogenase, *SRD5A1* steroid 5α-reductase 1, *SRD5A2* steroid 5α-reductase 2, *PND49* postnatal day 49. Full-length blots for (**B**) are presented in Supplementary Figs. S7 and S8.

To verify the effect of *Dapl1* ablation on testicular steroidogenesis, we compared mRNA expression levels of related proteins in the testes of wild-type and *Dapl1*-KO mice at postnatal day (PND) 49. In the mouse testis, increased mRNA expression of StAR was observed at PND49 due to *Dapl1* deletion (Fig. 3A). Western blotting further confirmed an increase in StAR protein expression (Fig. 3B). As cholesterol transport mediated by StAR is a crucial step in steroid formation, as expected, enzyme-linked immunosorbent assay (ELISA) results revealed that *Dapl1* deletion increased serum testosterone levels in mice (Fig. 3C). Given that testosterone is converted to dihydrotestosterone by steroid 5α-reductase (SRD5A), we examined the mRNA expression levels of SRD5A in the testis at PND49. Accordingly, we observed a significant increase in the mRNA expression levels of SRD5A1 and SRD5A2 (Fig. 3D). These findings indicated that *Dapl1* KO increased StAR expression in mouse testes, thereby increasing testosterone levels.

### *Dapl1* deletion may affect gonadotropin production in the hypothalamic-pituitary system through negative feedback regulation

**Figure 4:**
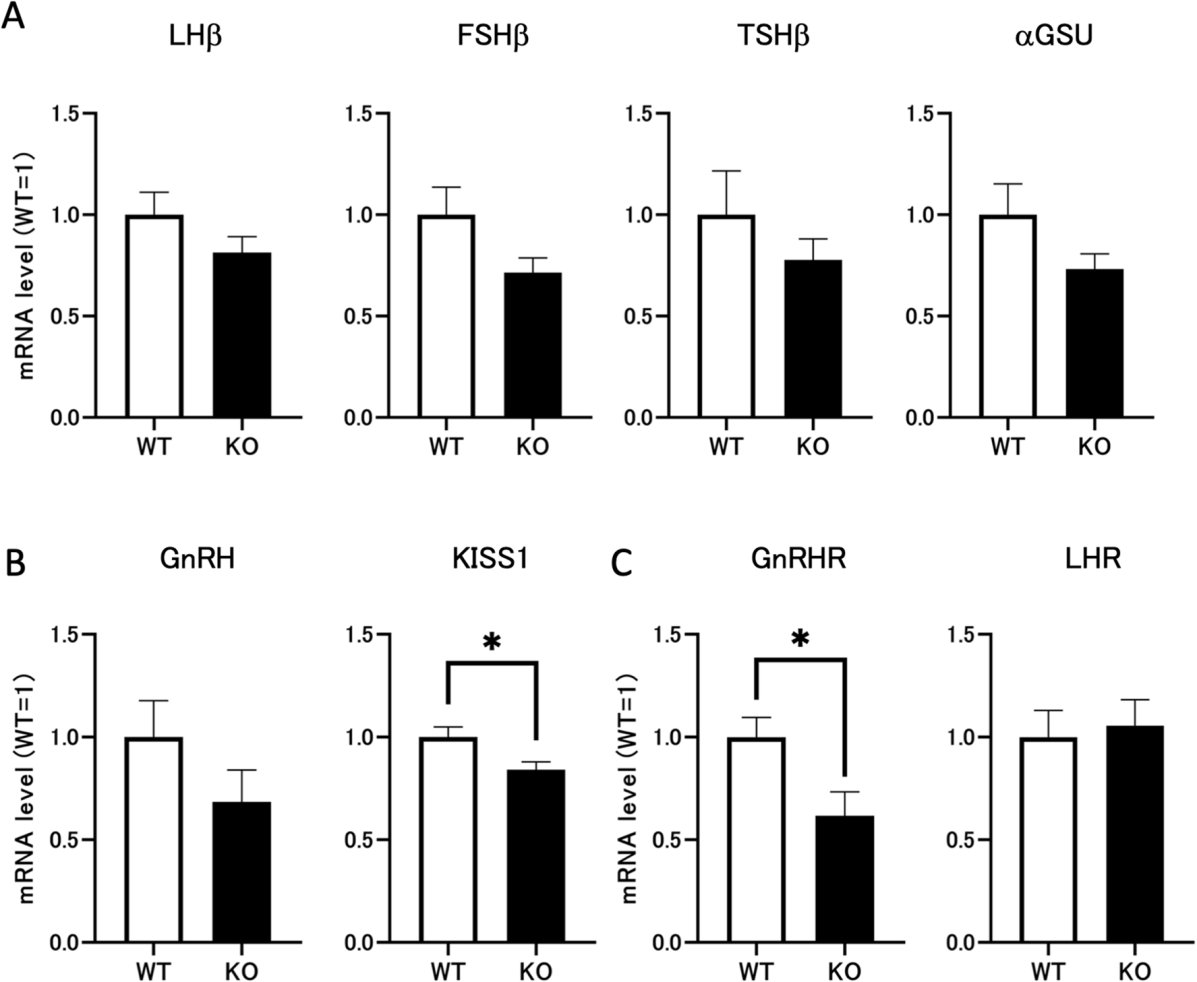
Effects of *Dapl1* ablation on the production of gonadotropins in the hypothalamic-pituitary system. (**A**) Effect of *Dapl1* ablation on the pituitary expression of mRNA coding for pituitary LHβ, FSHβ, TSHβ and αGSU in male mice at PND49. Each bar represents the mean ± S.E.M. of 11–12 mice. (**B**) Effect of *Dapl1* ablation on the hypothalamus gene-expression of mRNA coding for GnRH and KISS1 in male mice at PND49. Each bar represents the mean ± S.E.M. of 4–5 mice. (**C**) Effect of *Dapl1* ablation on the pituitary gene-expression of mRNA coding for GnRHR and the testicular steroidogenesis gene-expression of mRNA coding for LHR in male mice at PND49. Each bar represents the mean ± S.E.M. of 9–12 mice. Significantly different from the wild-type control: *p < 0.05. *DAPL1* death-associated protein-like 1, *LHβ* luteinizing hormone β subunit, *FSHβ* follicle-stimulating hormone β subunit, *TSHβ* thyroid-stimulating hormone β subunit, *αGSU* glycoprotein hormone α-subunit, *GnRH* gonadotropin-releasing hormone, *KISS1* kisspeptin, *GnRHR* gonadotropin-releasing hormone receptor, *LHR* luteinizing hormone receptor, *PND49* postnatal day 49.

Next, we compared mRNA expression levels of gonadotropin in the pituitary of wild-type and *Dapl1*-KO mice to determine whether the increased StAR expression is related to excessive gonadotropin secretion. In mice, *Dapl1* deletion induced a decreasing trend in the pituitary mRNA expression of the gonadotropin-related protein. (Fig. 4A). At PND49, GnRH in the male hypothalamus showed a downward trend, while mRNA levels of kisspeptin (KISS1) were significantly decreased (Fig. 4B). Hypothalamic GnRH is the central regulator of the HPG axis, and biosynthesis and release of GnRH in the hypothalamus are directly regulated by arginine vasopressin (AVP) from the suprachiasmatic nucleus and KISS1 from the anteroventral periventricular nucleus^17^. Finally, we examined mRNA expression levels of the GnRH receptor (GnRHR) in the pituitary gland and LHR in the testis to determine the initiation point of abnormal hormone secretion in the HPG axis following *Dapl1* deletion. As expected, *Dapl1* KO significantly reduced the mRNA expression of GnRHR in the pituitary but did not affect the expression of LHR in the testis (Fig. 4C). Based on the above results, we speculated that an important factor in the HPG axis hormone secretion disorder, as caused by *Dapl1* KO, is the outcome of negative feedback regulation induced by increased endogenous testosterone.

### DAPL1 inhibits sex steroid hormone synthesis in the mouse Leydig cells

**Figure 5:**
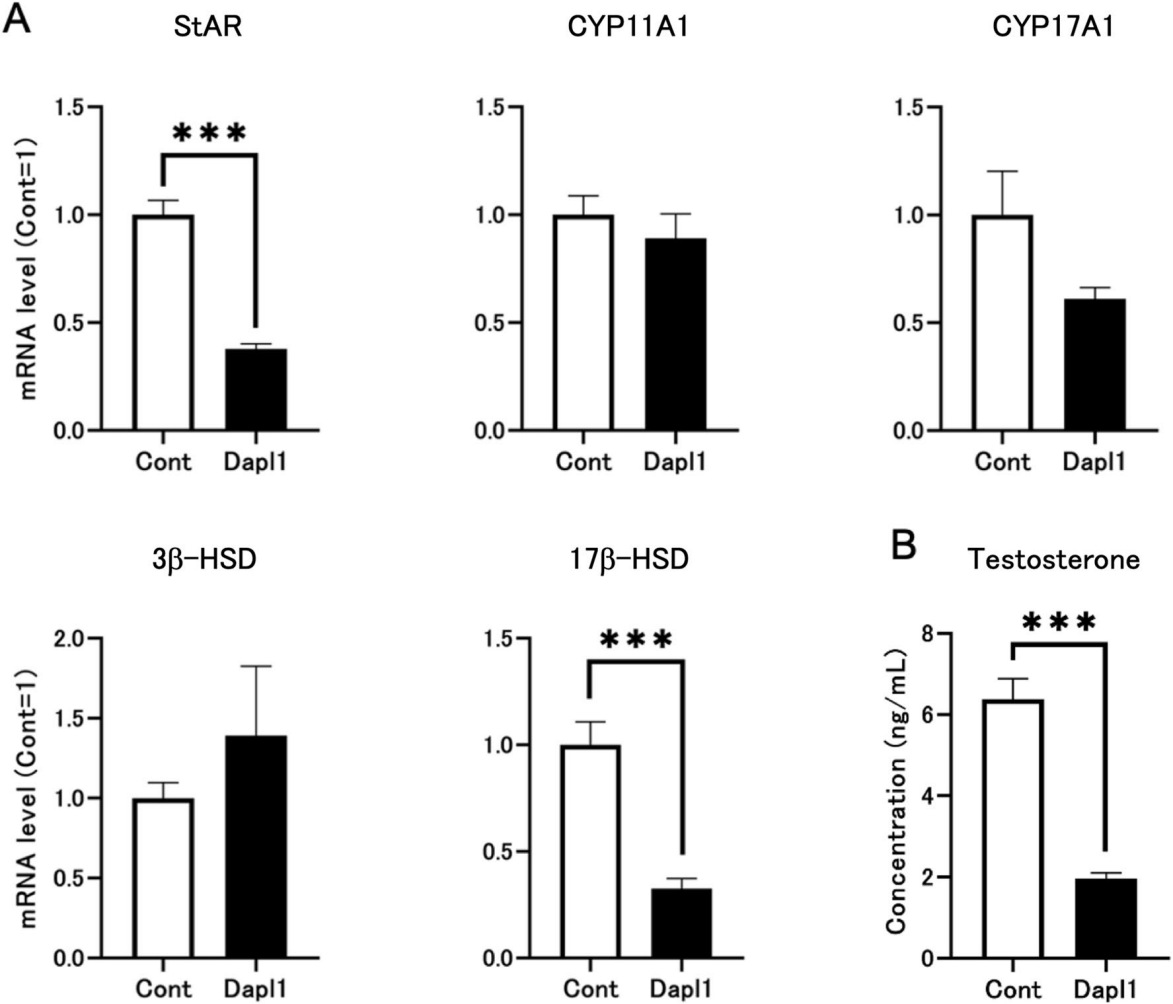
Effects of *Dapl1* transfection on neutral steroid hormone synthesis in I-10 cells. (**A**) Effect of *Dapl1* transfection on the testicular steroidogenesis gene-expression of mRNA coding for StAR, CYP11A1, CYP17A1, 3β-HSD, and 17β-HSD in I-10 cells. I-10 cells seeded in 6-well plates were transfected with pcDNA3.1-mouse *Dapl1* (2 µg/well). For the control, cells were transfected with the empty pcDNA-3.1(−)-hygro vector. Each bar represents the mean ± standard error of the mean (S.E.M.) of 6 samples. (**B**) Effect of *Dapl1* transfection on level of testosterone in I-10 cell culture medium. Each bar represents the mean ± S.E.M. of 8 samples. Significantly different from the control I-10 cells: ***p < 0.001. *DAPL1* death-associated protein-like 1, *StAR* steroidogenic acute regulatory protein, *CYP* cytochrome P450, *3β-HSD* 3β-hydroxysteroid dehydrogenase, *17β-HSD* 17β-hydroxysteroid dehydrogenase, *SRD5A1* steroid 5α-reductase 1, *SRD5A2* steroid 5α-reductase 2, *PND49* postnatal day 49.

Next, to determine whether DAPL1 expression in the testis impacts the steroid synthesis, we used I-10 mouse testis-derived tumor cells to perform a confirmatory in vitro experiment. Interestingly, the mRNA expression level of DAPL1 was considerably lower in I-10 cells than in wild-type mouse testis. We used plasmids to transfect *Dapl1* cDNA into I-10 cells to investigate whether high *Dapl1* expression affects steroid synthesis in Leydig cells. Compared with control cells, the mRNA expression levels of StAR and 17β-hydroxysteroid dehydrogenase (17β-HSD) were significantly reduced in *Dapl1*-transfected I-10 cells (Fig. 5A). Subsequently, the medium was tested by ELISA, and it was observed that DAPL1 overexpression induced by transient transfection significantly inhibited testosterone production in I-10 cells (Fig. 5B). These in vitro experimental results highlighted that DAPL1 inhibits the expression of StAR and 17β-HSD in Leydig cells, thereby inhibiting testosterone production, consistent with the results of the above-mentioned animal experiments using *Dapl1*-KO mice.

### *Dapl1* deletion stimulates the PKA system and CREB/CREM pathway in mouse testes at PND49

**Figure 6:**
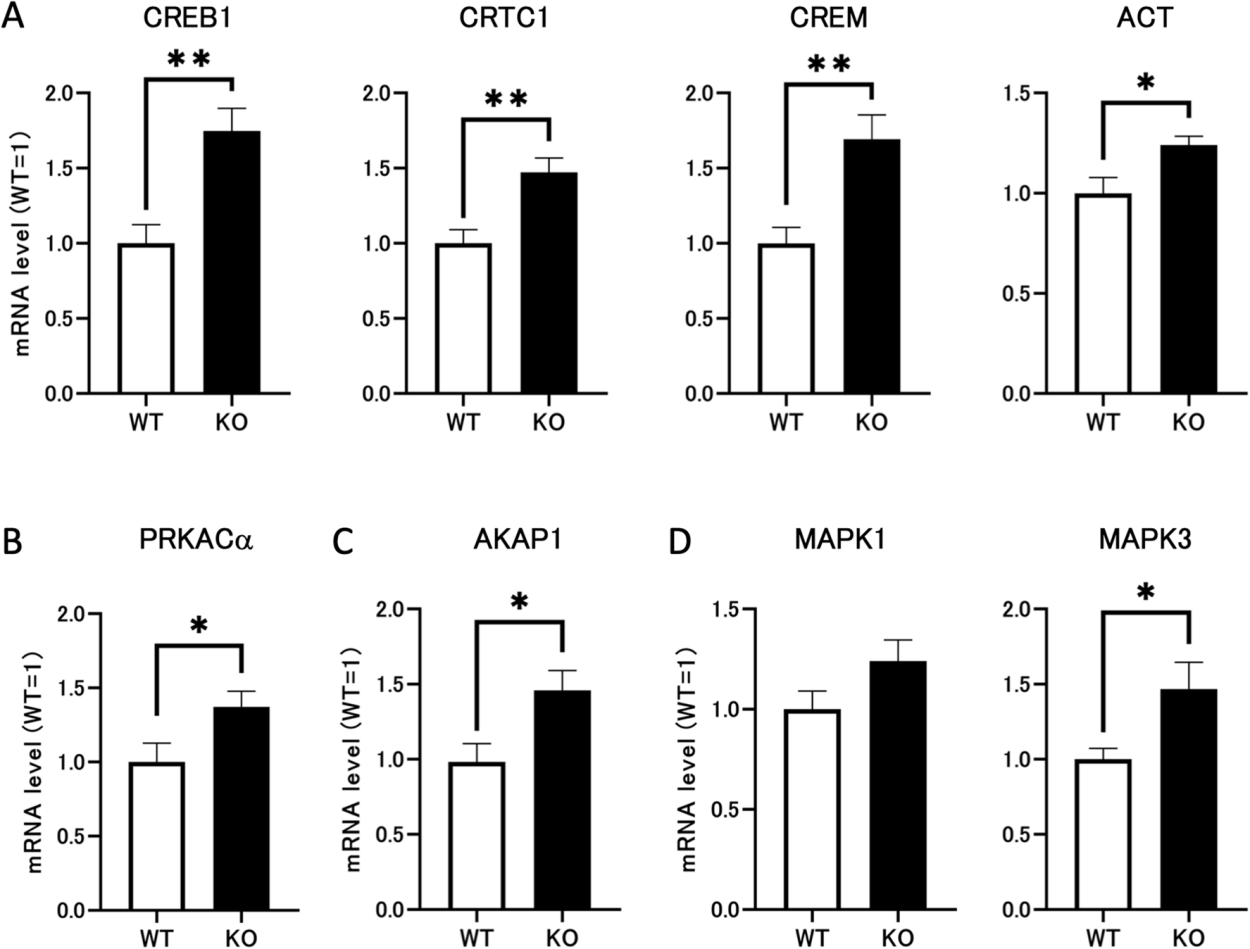
Effects of *Dapl1* deletion on the PKA system and CREB/CREM pathway in mouse testes at PND49. (**A**) Effect of *Dapl1* ablation on the testicular CREB/CREM pathway gene-expression of mRNA coding for CREB1, CRTC1, CREM, and ACT in mice at PND49. Each bar represents the mean ± S.E.M. of 9–12 mice. (**B**) Effect of *Dapl1* ablation on the testicular PKA system gene-expression of mRNA coding for PRKACα in mice at PND49. Each bar represents the mean ± S.E.M. of 9–12 mice. (**C**) Effect of *Dapl1* ablation on gene-expression of mRNA coding for AKAP1 in mice testes at PND49. Each bar represents the mean ± S.E.M. of 11–12 mice. (**D**) Effect of *Dapl1* ablation on the testicular MAPK/ERK pathway gene-expression of mRNA coding for MAPK1 and MAPK3 in mice at PND49. Each bar represents the mean ± S.E.M. of 11–12 mice. Significantly different from the wild-type control: *p < 0.05, **p < 0.01. *DAPL1* death-associated protein-like 1, *CREB* cyclic AMP-response element-binding protein; *CREM* CRE modulator protein; *CRTC1* CREB regulated transcription coactivator 1; *ACT* activator of CREM in testis; *PRKACα* protein kinase, cAMP-dependent, catalytic, α; *AKAP1* A-kinase anchor protein 1; *MAPK* mitogen-activated protein kinase; *PND49* postnatal day 49.

We next attempted to elucidate the upstream mechanism underlying increased testicular StAR expression induced by *Dapl1* deletion. Accordingly, we compared mRNA expression levels of CREB1 and CREM in the CREB/CREM pathway, as well as their co-activators, CREB-regulated transcription coactivator 1 (CRTC1) and activator of CREM in testes (ACT), in the testes of wild-type and *Dapl1* KO mice at PND49. As expected, *Dapl1* deletion significantly increased mRNA expression levels of CREB1 and CREM in the mouse testis at PND49; additionally, mRNA expression of CRTC1 and ACT were significantly increased (Fig. 6A). PKA is an important upstream protein of the CREB/CREM pathway. Among the three isoforms of its catalytic subunits, PRKACα is considered the predominant isoform and is found to be expressed in most tissues^18^. In the testes of adult *Dapl1* KO mice, the mRNA expression level of PRKACα was significantly increased (Fig. 6B). A-kinase anchoring protein 1 (AKAP1**)** can induce PKA accumulation in mitochondria and affect the mitochondrial translation of StAR mRNA^19^. *Dapl1* deletion significantly increased the mRNA expression of APAK1 in the mouse testis (Fig. 6C). Another important upstream factor of the CREB/CREM pathway is MAPK3/1 (also known as ERK1/2)^20–22^. Activated MAPK3/1 and PKA activate the transcription factor CREB by increasing its expression and phosphorylation^23^. In the testes of adult *Dapl1*-KO mice, the mRNA expression levels of MAPK1 showed an upward trend. Similarly, the mRNA expression level of MAPK3 was significantly increased (Fig. 6D). These results indicate that *Dapl1* deletion can cause abnormal activation of the PKA and CREB/CREM pathways. It is reasonable to suppose that these results are directly related to the increased StAR expression.

### DAPL1 reduces StAR expression by inhibiting the CREB/CREM pathway and PKA system in I-10 cells

**Figure 7:**
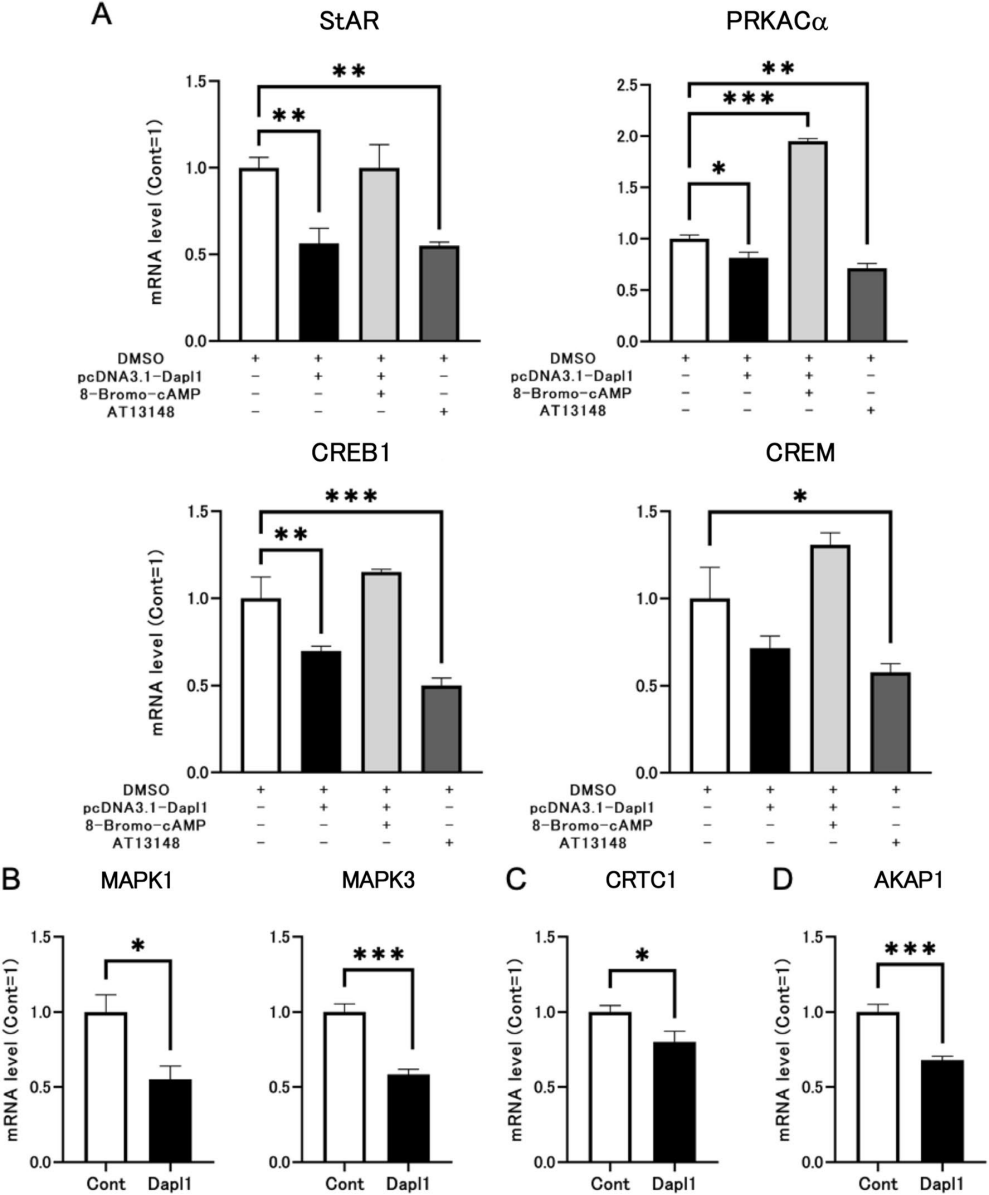
Effects of *Dapl1* transfection on the PKA-CREB/CREM-StAR pathway in I-10 cells. (;) The effect of *Dapl1* transfection and PKA modulating agents on the PKA system and CREB/CREM pathway gene-expression of mRNA coding for StAR, PRKACα, CREB1, and CREM in I-10 cells. We used an empty plasmid to transfect the "pcDNA3.1-*Dapl1* (−)" group of cells, and used the pcDNA3.1-mouse *Dapl1* expression plasmid to transfect the "pcDNA3.1-*Dapl1* (+)" group of cells. After transfection, the samples were incubated for 48 h, and the related drugs dissolved in DMSO were added. Among them, only the “pcDNA3.1-*Dapl1* (−)” group sample with DMSO solvent was added as a control group. Each bar represents the mean ± S.E.M. of 3–4 samples. (**B**) Effect of *Dapl1* transfection on the MAPK/ERK pathway gene-expression of mRNA coding for MAPK1 and MAPK3 in I-10 cells. Details of transfection are described in the legend to Fig. 5 and the Material and Methods. Each bar represents the mean ± S.E.M. of 5–6 samples. (**C**) Effect of *Dapl1* transfection on the CREB/CREM pathway gene-expression of mRNA coding for CRTC1 in I-10 cells. Each bar represents the mean ± S.E.M. of 5–6 samples. (**D**) Effect of *Dapl1* transfection on the gene-expression of mRNA coding for AKAP1 in I-10 cells. Each bar represents the mean ± S.E.M. of 5–6 samples. Significantly different from the control I-10 cells: *p < 0.05, **p < 0.01, ***p < 0.001. *DAPL1* death-associated protein-like 1, *StAR* steroidogenic acute regulatory protein, *PKA* protein kinase A, *CREB* cyclic AMP-response element-binding protein, *CREM* CRE modulator protein, *CRTC1* CREB-regulated transcription coactivator 1, *AKAP1* A-kinase anchor protein 1, *MAPK* mitogen-activated protein kinase.

Herein, we performed in vitro experiments employing I-10 cells to verify whether the abnormal activation of the PKA system and CREB/CREM pathway can directly result in increased StAR expression following *Dapl1* deletion in the mouse testis. We hypothesized that DAPL1 regulates the transcription of related proteins in the CREB/CREM pathway by inhibiting PKA expression in Leydig cells, thereby affecting StAR expression. First, we divided the cells into four groups and seeded them onto six-well plates. Forty-eight hours after transfection, 8-bromo-cAMP and TA13148 were employed as the activator and inhibitor of PKA, respectively, to treat the corresponding cell groups. In preliminary experiments, we determined optimal concentrations required to observe effects on mRNA expression of StAR and PRKACα in I-10 cells. The concentrations of 8-bromo-cAMP and AT13148 were established as 5 µM and 100 nM, respectively (Figs. S1 and S2). After 24 h of incubation, the cells of each group were collected, and the mRNA expression of related genes was evaluated. In mouse *Dapl1*-transfected I-10 cells, mRNA expression levels of StAR, PRKACα, and CREB1 were significantly reduced when compared with those in the control group (Fig. 7A). On adding 8-bromo-cAMP to *Dapl1*-transfected I-10 cells, expression levels of PRKACα and CREB1 were restored to normal levels, while the altered trend of CREM mRNA expression was similar to that of StAR and CREB1; however, no statistical significance was observed (Fig. 7A). Following the addition of AT13148 to control plasmid-transfected cells, mRNA expression levels of StAR, PRKACα, CREB1, and CREM were significantly decreased. Simultaneously, it was observed that transient *Dapl1*-transfection, as the primary condition employed, and TA13148 presented approximately similar effects on I-10 cells (Fig. 7A). Next, we verified the results of in vivo experiments by comparing mRNA expression levels of genes of interest in control group I-10 cells and *Dapl1*-transfected I-10 cells. We observed *Dapl1* transfection significantly reduced mRNA expression levels of CRTC1, APAK1, and MAPK1/3 in I-10 cells (Fig. 7B–D); these findings were consistent with results of previous animal experiments (Fig. 6A,C,D). These results indicate that one pathway via which DAPL1 regulates StAR expression in Leydig cells could involve mediation via the PKA and CREB/CREM pathways.

## Discussion

In the present study, *Dapl1*-KO mice were employed to investigate the physiological functions of DAPL1. We observed that DAPL1 mRNA was highly expressed in mouse testes, and *Dapl1* KO increased the transcription level of PRKACα in the PKA system and MAPK3/1 in the MAPK/ERK pathway in mouse testes, thereby increasing the expression of CREB1 and CREM in the CREB/CREM pathway, as well as their co-activators CRTC1 and ACT, subsequently promoting the transcription level of StAR. Simultaneously, *Dapl1* KO increases the expression of AKAP1 in mitochondria, which leads to increased mitochondrial PKA recruitment, resulting in more efficient translation and phosphorylation of StAR^19^, and ultimately an increase in serum testosterone levels. In vitro experiments revealed that DAPL1 overexpression reduced testosterone production in I-10 testicular tumor cells. This overexpression responds to a mechanism in which DAPL1 inhibits the expression of key protein mRNA in the PKA and CREB/CREM pathways, thus inhibiting StAR expression. Similarly, we observed that when an appropriate concentration of a PKA activator was added, the mRNA expression of StAR returned to the same level as that observed in the control group. Collectively, the in vivo and in vitro experimental findings demonstrated that DAPL1 plays a vital role in the steroidogenic system of the testis.

Furthermore, we compared the mRNA expression levels of DAPL1 in different tissues of adult male mice and observed that DAPL1 mRNA expression was considerably higher in the eyeball, pituitary gland, and testis than in other investigated tissue specimens. Our data are consistent with reports revealing that DAPL1 inhibits cellular hyperproliferation in the retinal pigment epithelium of adult mice^14,15^, and also with a previous report from our laboratory, which suggested that reducing DAPL1 expression will decrease the number of GH-positive cells in the pituitary gland of fetal rats^24^. To the best of our knowledge, no previous report has evaluated DAPL1 in the testis. Our study revealed, for the first time, the importance of DAPL1 in the testicular steroid production system, demonstrating that *Dapl1* deletion affects the gonadotropin secretion system in the HPG axis of male adult mice. Given that current reports on DAPL1 present varying effects on different tissues, cells, and ages^13–15,24–26^, as well as high expression levels of DAPL1 mRNA in the pituitary gland of adult mice (Fig. 1A), we cannot rule out the hypothesis that *Dapl1* knockout directly affects the gonadotropin-secreting system in the hypothalamic-pituitary axis. Therefore, further research is necessary to comprehensively elucidate this possibility. However, based on current experimental results, we strongly believe that a negative feedback response to high serum testosterone levels mediates the effect of *Dapl1* KO on the gonadotropin-secreting system in the hypothalamic-pituitary axis of adult male mice. This is supported by an in-depth analysis revealing that *Dapl1* KO leads to a significant decrease in the hypothalamic expression of KISS1 mRNA in adult mice (Fig. 4B), which is consistent with previous reports indicating that dihydrotestosterone inhibits the mRNA expression of KISS1 during the negative feedback regulation of androgens^27^.

Moreover, we observed that in I-10 cells derived from mouse testicular Leydig cell tumors, the mRNA expression level of DAPL1 was markedly lower than that in the testis (Fig. S3); this could be attributed to the low expression of DAPL1 in Leydig cells in contrast to the high expression observed in other cell types within the testis. We used a single-cell sequencing dataset to explore the testicular localization of DALP1 (https://cells.ucsc.edu/ at 18/6/2021)^28^. The data revealed that DAPL1 is widely expressed in adult human testes and is highly expressed in germ cells^28,29^, which supports the above-mentioned hypothesis. In addition, testicular histopathological observations of *Dapl1*-KO mice showed that the seminiferous tubule cell density of *Dapl1*-KO mice was often higher than that of wild-type mice (Fig. S4). We speculate that the cause of this result is related to the direct action of DAPL1 on the cells in seminiferous tubules, besides the excess secretion of testosterone. The data we currently have are not sufficient to prove that DAPL1 only affects the PKA and CREB/CREM pathways in Leydig cells and does not affect the same protein in other types of testicular cells. This requires in vitro experiments using other types of testicular cells for future studies. Nevertheless, StAR is thought to be exclusively present in Leydig cells.

It has been reported that *DAPL1* expression is suppressed in breast cancer tissues^25^; accordingly, we can alternatively postulate that when compared with normal Leydig cells, DAPL1 expression in Leydig cell tumors is inhibited. Additionally, on incorporating AT13148 into I-10 cells, mRNA expression of DAPL1 significantly increased (Fig. S5). This result seems to provide some clues to the above hypothesis. It is worth mentioning that AT13148 not only inhibits PKA but also inhibits a variety of AGC kinases, including AKT, p70S6K, SGK, and ROCK. AT13148 is a new type of oral multi-AGC kinase inhibitor with powerful pharmacodynamics and anti-tumor activities^30^. During in vitro experiments, *Dapl1* transfection significantly reduced PRKACα mRNA expression in I-10 cells when compared with controls, consistent with reports demonstrating that the DAPL1 affects PRKACα expression levels during the development and progression of invasive breast carcinoma^25^. The addition of 8-bromo-cAMP increased mRNA expression of PRKACα by two-fold when compared with the control group; simultaneously, the mRNA expression of StAR was restored to the same level as in the control group in *Dapl1*-transfected I-10 cells. Accordingly, we speculate that in addition to the PKA and CREB/CREM pathways, DAPL1 also affects the transcription of the *Star* gene through alternate pathways. We also observed that an appropriate AT13148 concentration and *Dapl1* transfection essentially had the same inhibitory effect on related protein mRNA in I-10 cells, which also clarifies the above postulation. The specific molecular mechanism via which DAPL1 inhibits the expression of PRKACα and MAPK3/1 needs to be elucidated in a future study.

In conclusion, our work provides evidence that DAPL1 plays a critical regulatory role in the testicular steroid system and highlights symptoms associated with dysregulated endogenous testosterone levels.

## Materials and methods

### Reagents and antibodies

8-Bromo-cAMP and AT13148, used for in vitro testing, were purchased from Selleck Chemicals (Houston, TX, USA). Mouse anti-rabbit StAR polyclonal antibody and mouse anti-β-actin monoclonal antibody were purchased from Santa Cruz Biotechnology Inc. (1:1000, Dallas, TX, USA) and BioVision Inc. (1:2500, Mountain View, CA, USA), respectively. Horseradish peroxidase-labeled anti-mouse IgG was provided by GE Healthcare (1:5000, Chicago, IL, USA). All other reagents were of the highest commercially available grade.

### Animals and treatments

All animal experiments were approved by the Institutional Animal Care and Experimental Committee of Kyushu University. All methods were carried out in compliance with National Institutes of Health (NIH) guidelines for the care and use of laboratory animals. This study was also carried out in compliance with the ARRIVE guidelines. Female (6-week-old) C57BL/6 J mice were purchased from CLEA Japan Inc. (Tokyo, Japan). CRISPR/Cas9 mediated *Dapl1* KO mice (19-week-old), as well as their heterozygous offspring (6-week-old), were generated by Unitech (Chiba, Japan). Cas9 mRNA was prepared from linear DNA templates using CAS500A-1 Transfection-ready Cas9 SmartNuclease mRNA (System Biosciences, Palo Alto, CA, USA) and generated using CAS510A-1 Linearized T7 gRNA SmartNuclease Vector Kit (System Biosciences), according to the manufacturer’s instructions. The following sequences were used for sgRNA synthesis: sgRNA1, ACTTTCGACCTCCCTACGC; sgRNA2, GAACCAGGTGACCCATTTCT. Cas9 mRNA and sgRNAs were microinjected into fertilized embryos of C57BL/6 J mice. Deletion mutations in exon 2 and exon 3 of the mouse *Dapl1* gene sequence were identified in the offspring. A purchased F1 heterozygous *Dapl1* knockout mouse was selected for subsequent experiments. *Dapl1*-KO type (F1) mice were backcrossed with C57BL/6 J mice to generate the F2 generation. Similarly, male *Dapl1*-KO type (F2) mice were backcrossed with C57BL/6 J mice to generate the F3 generation. Animals were housed under a 12 h light/12 h light–dark cycle in an air-conditioned room and provided with food and tap water ad libitum. Wild-type and *Dapl1*-KO mice were obtained by mating male and female *Dapl1*-heterozygous mice (Fig. 2C). KO deletion of bases, including exons 2 and 3 (Fig. 2A), were confirmed by direct sequencing. PCR was employed to genotype newborn pups before weaning at 3-weeks of age using ear-derived genomic DNA (Fig. 2B); primers used for genotyping were as follows: forward, 5′-GCTCTGGCTTCCTTAGTTGTTTT-3′; reverse, 5′-TTTAGAGGCACTAAGGCTTTTGG-3′. From wild-type and the *Dapl1*-KO mice, both tissue and blood were collected at PND49. The mice were sacrificed under carbon dioxide euthanasia between 9 and 11 a.m., and the hypothalamus, pituitary gland, and testes were snap-frozen in liquid nitrogen and maintained at − 80 °C until use. Serum samples were prepared via blood centrifugation at 3000×*g* for 15 min at 4 °C, snap-frozen, and stored at − 80 °C prior to use.

### Cell culture

I-10 cells (JCRB9097, Passage number: 42; P42) originating from mouse Leydig testicular tumor cells were purchased from the Japanese Cancer Research Resources Bank (Osaka, Japan)^31^. The cells were maintained in HAM’S/F-10 medium (Hyclone Laboratories, Logan, UT, USA) supplemented with 15% horse serum (Sigma-Aldrich, St. Louis, MO, USA) and 2.5% fetal bovine serum (PAA Laboratories, Inc., Etobicoke, Canada) in a 5% CO_2_ humidified incubator at 37 °C. The reagents (8-bromo-cAMP and AT13148) used in the in vitro cell experiment were dissolved in dimethyl sulfoxide (DMSO) at concentrations of 50 mM and 10 mM, respectively, to obtain a stock solution. During the experiment, relevant drugs were diluted with DMSO and added to the culture medium. The cells (P54) were transfected and incubated for 48 h; then, the corresponding drugs were diluted in DMSO and added to the medium for further incubation for 24 h. During this period, the DMSO concentration in the culture medium of each cell sample was 0.1% (v/v). After incubation, cells and the culture medium were harvested for RT-PCR and enzyme immunoassays.

### Plasmid construction and transfection

A pcDNA3.1-mouse *Dapl1* expression plasmid was constructed. *Dapl1* cDNA was amplified by nested RT-PCR from the total RNA derived from the corneal epithelium of C57BL/6 J mice. The primer sequences used for the first RT-PCR were as follows: *Dapl1* forward m*Dapl1*(− 56, − 29)F 5′-AGTTACAACTGGCACTCAGCCTCAGAG-3′, reverse m*Dapl1*(425, 399)R 5′-AGATTGTGGGAGAGTTGACCAGGTGGC-3′. The following primers were employed for the second nested PCR: forward XhoI-m*Dapl1*(− 11, + 10)F 5′-CCGCTCGAGACACAGGCAC TATGGCAAAC G-3′, reverse BamHI-m*Dapl1*(390, 366)R 5′-CGGGATCCCTGTCCTGGTCTAACATTTT CGAGG-3′. The restriction sites XhoI and BamHI are respectively underlined. After amplification using KOD DNA polymerase (Toyobo, Osaka), the products were digested with XhoI and BamHI and subcloned into the pCDNA3.1(−)-Hygro vector. The inserted sequences were verified by DNA sequencing and digested by restriction endonucleases (XhoI and BamHI). Cells (P48 and P53) were seeded in six-well plates (1 × 10^6^ cells/well) and cultured for 24 h. Transfection with pcDNA3.1-mouse *Dapl1* was performed using polyethylenimine HCl MAX (Linear, MW 40000, Transfection Grade) (PEI) (Polysciences, Inc., Warrington, PA). PEI was dissolved into purified water at 1 mg/mL, and sterilized with a filter for use in transfection.

### RT-PCR

mRNA expression was quantified by real-time RT-PCR according to the method described in our previous report^32,33^. Briefly, total RNA was extracted from the testes and I-10 cells using RNeasy kits (QIAGEN GmbH, Hilden, Germany). The RNA obtained was treated with gEraser (TaKaRa-bio, Shiga, Japan) to digest contaminating genomic DNA and then reverse transcribed to cDNA. Target mRNAs were amplified with Fast SYBR Green Master Mix (Thermo Fisher Scientific, Inc., Waltham, MA, USA), using the StepOnePlus real-time PCR system (Thermo Fisher Scientific). The primer design and PCR conditions are described in Table S1 and previous reports^32,33^. The relative mRNA expression was determined using the standard curve method. The amount of quantified target mRNA was normalized to β-actin mRNA and is shown as a ratio to the control.

### Immunoblotting

The expression of testicular StAR and β-actin proteins was analyzed by immunoblotting according to a previously described method with minor modifications^32,34^. Briefly, frozen testes samples were added to cold potassium phosphate buffer (pH 7.4; 0.25 M sucrose, 1 mM dithiothreitol, and protease inhibitor cocktail [Roche Diagnostics GmbH, Mannheim, Germany]) and homogenized. Subsequently, the samples were centrifuged at 1000×*g* for 10 min, and resulting supernatants were centrifuged at 9000×*g* for 20 min. Next, samples underwent a 10% sodium dodecyl sulfate–polyacrylamide gel electrophoresis, and proteins were detected using anti-StAR (1:1000) and anti-β-actin (1:2500) IgGs as primary antibodies. The amount of protein used for electrophoresis was 50 μg for both StAR and β-actin.

### Enzyme-linked immunosorbent assay

Testosterone levels were determined by ELISA using commercial kits (Cayman Chemical, Ann Arbor, MI, USA). The serum was diluted 20-fold, and the cell culture medium was diluted 50-fold with the buffer supplied in the kits.

### Statistical analysis

Statistical differences between any two groups were compared using Student’s *t* test. For comparing multiple groups, statistical differences were calculated by one-way analysis of variance with a post-hoc test (Dunnett’s test [cell culture experiments]) using GraphPad Prism version 8.0 (GraphPad Software, San Diego, CA). Statistical significance was set at *p* < 0.05.

## Supplementary Information

Supplementary Information.
