# Supplementary material for: DAPL1 is a novel regulator of testosterone production in Leydig cells of mouse testis
Source: Sci Rep. 2021 Sep 17;11:18532. doi: 10.1038/s41598-021-97961-6 (PMC8448858; doi:10.1038/s41598-021-97961-6)
Supplement: Supplementary file 1 — Supplementary Information. [file 41598_2021_97961_MOESM1_ESM.pdf]

DAPL1 is a novel regulator of testosterone production in Leydig cells of mouse testis

Hong-bin Chen[1,2], Jorge Carlos Pineda Garcia[1,2], Shinako Arizono[1,2], Tomoki Takeda[1,4], Ren-shi Li[1,3], Yukiko Hattori[1], Hiroe Sano[1,2], Yuu Miyauchi[2], Yuko Hirota[2], Yoshitaka Tanaka[2], Yuji Ishii[1,2]

[1] Laboratory of Molecular Life Sciences, Graduate School of Pharmaceutical Sciences, Kyushu University, Fukuoka, Japan

[2] Division of Pharmaceutical Cell Biology, Graduate School of Pharmaceutical Sciences, Kyushu University, Fukuoka, Japan

[3]Sino-Jan Joint Lab of Natural Health Products Research, School of Traditional Chinese Medicines, China Pharmaceutical University, Nanjing, China

[4]Division of Experimental, Japan Bioassay Research Center, Japan Organization of Occupational Health and Safety, Hadano, Japan

Scientific Reports

Table S1. Primers used in the PCR for the quantification of mRNA

Targets	Forward (5' -> 3')<br>Reverse (5' -> 3')	Size of product (bp)	Accession No.[a]
**DAPL1**	GGCGTTTTGGAAAGACACAC<br>CCGTCAGAGCATCCAACATC	94	NM_029723
**StAR**	GACCTTGAAAGGCTCAGGAAGAAC<br>GCACAGATTGGTGCCTTAATCC	135	NM_011485
**CYP11A1**	AAGACCTGGAAGGACCATGC<br>CACCAGGGTACTGGCTGAAG	103	NM_019779
**CYP17A1**	CTGCAGTGATTGTCGGTCAC<br>GACAAGAGGCCTAGAGTCAC	112	NM_013046
**3β-HSD**	GAGGAGATCAGCATCCAGAC<br>ACAGATGACAGTGGGAGCTG	98	NM_008293
**17β-HSD**	AGTTGGCCAGACATGGACTC<br>ACACAGCTTCCAGTGGTCCTC	100	NM_008291
**SRD5A1**	GTAACCCATCCCTGTTTCCTG<br>CAGTGCAAAGCCACACCACT	104	NM_175283
**SRD5A2**	CACAGACATGCGGTTTAGCG<br>CAGGAAATTGGCTCCAGACAC	163	NM_053188
**LHβ**	TGGCCGCAGAGAATGAGTTC<br>ACTCGGACCATGCTAGGACA	85	NM_008497
**FSHβ**	GGAGAGCAATCTGCTGCCAT<br>GCCGAGCTGGGTCCTTATAC	150	NM_008045
**TSHβ**	CACCATCTGTGCTGGGTATTG<br>CATCCTGGTATTTCCACCGTTC	129	NM_009432
**αGSU**	CAAAAAGTCCAGAGCTTGCAG<br>ATATGCAGGAACATGGACAGC	89	NM_009889
**GnRH**	CCTACCAACGGAAGCTCGAG<br>GCAGCCTTCCAAACACACAG	111	NM_008145
**GnRHR**	TGCTCTCACGCGAGTCCTTC<br>GGAGGTAGCGAATGCGACTG	118	NM_010323
**KISS1**	CCTCTGTGTCGCCACCTATG<br>GCTTGCTCTCTGCATACCGC	125	NM_178260
**LHR**	CTCGCCCGACTATCTCTCAC<br>ACGACCTCATTAAGTCCCCTG	77	NM_013582
**CREB1**	GCCATCAGTTATCCAGTCTC<br>TTCCTGTAGGAAGGCCTCCTTG	145	NM_133828
**CRTC1**	TCATGGGCTTGTGGACAGAG<br>CAGGGCAGAGTCAGAGTTGGT	175	NM_001004062

**CREM**	GCAGGATCGAAGTGTAACACG<br>GAATACCAGGCACATCAGAGG	124	NM_001374833
**FHL5**	AAGGATGATCGCCTGCTGTG<br>GTTCCTATTGGCTGTCGGCA	167	NM_021318
**PRKACα**	GAGAGTGGGAACCACTACGC<br>GACCAGGAACGGGAAGTTGAC	123	NM_008854
**AKAP1**	TGTCAGACTTAGGGACCGAG<br>CTTGGTAAGCCCGCAACAGCT	113	NM_009648
**MAPK1**	TCAAGCCTTCCAACCTCCTG<br>CTCTGTACCAACGTGTGGCT	135	NM_001357115
**MAPK3**	GAGGGAGATCCAGATCTTGC<br>CTGTCTCCATGAGGTCCTGA	125	NM_011952
**β-actin**	GATTACTGCTCTGGCTCCTA<br>TCCTGCTTGCTGATCCAC	135	NM_007393

[a] The GenBank accession numbers are shown.

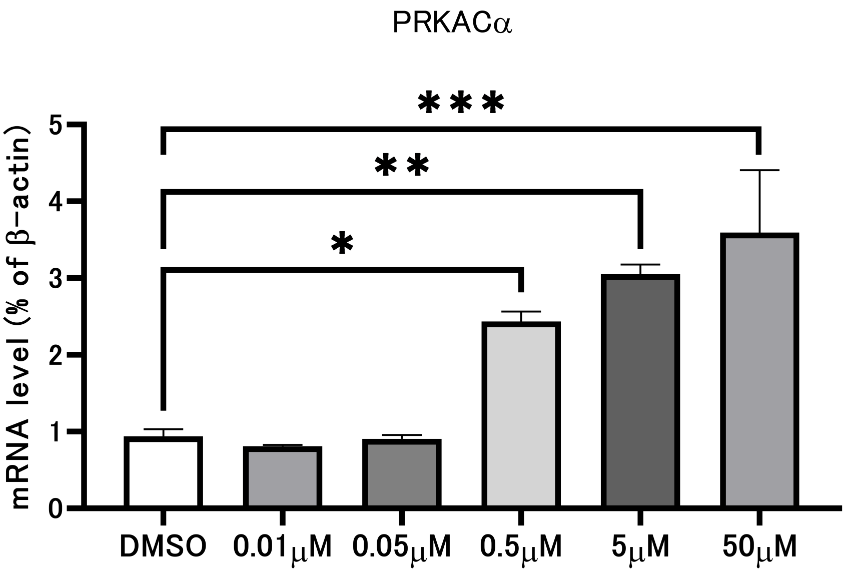

Fig. S1. Effect of different concentrations of 8-bromo-cAMP on the gene-expression of mRNA coding for PRKACα in I-10 cells. Each bar represents the mean ± S.E.M. of 3 samples. Significantly different from the DMSO group: *$p < 0.05$, **$p < 0.01$, ***$p < 0.001$. DMSO, dimethyl sulfoxide.

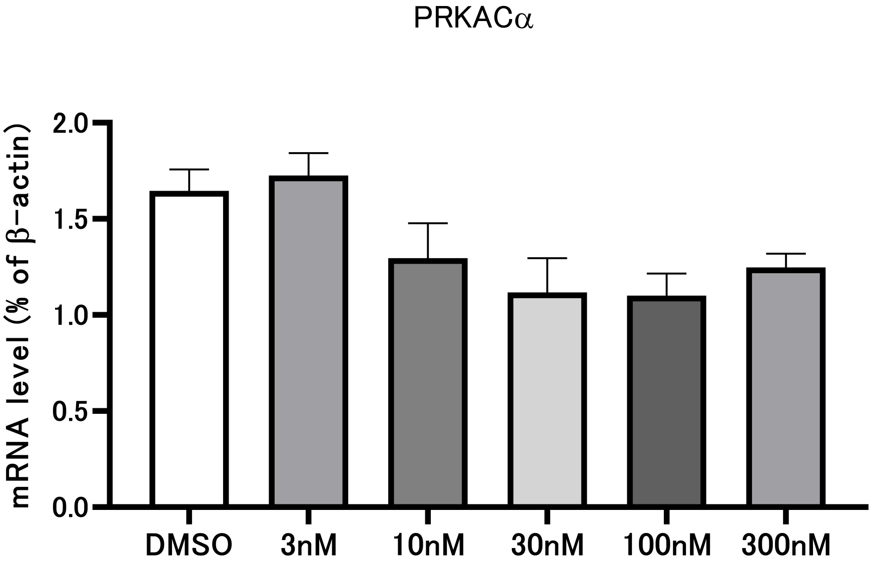

Fig. S2. Effect of different concentrations of TA13148 on the gene-expression of mRNA coding for PRKACα in I-10 cells. Each bar represents the mean ± S.E.M. of 3 samples.

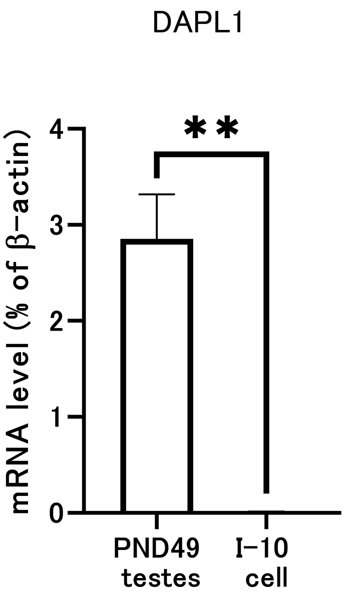

Fig. S3. Comparison of DAPL1 mRNA expression levels in testes of PND49 mice and I-10 cells. Each bar represents the mean ± S.E.M. of 4-6 samples. Significantly different from the testes group: **$p < 0.01$. DAPL1, death-associated protein-like 1; PND49, postnatal day 49.

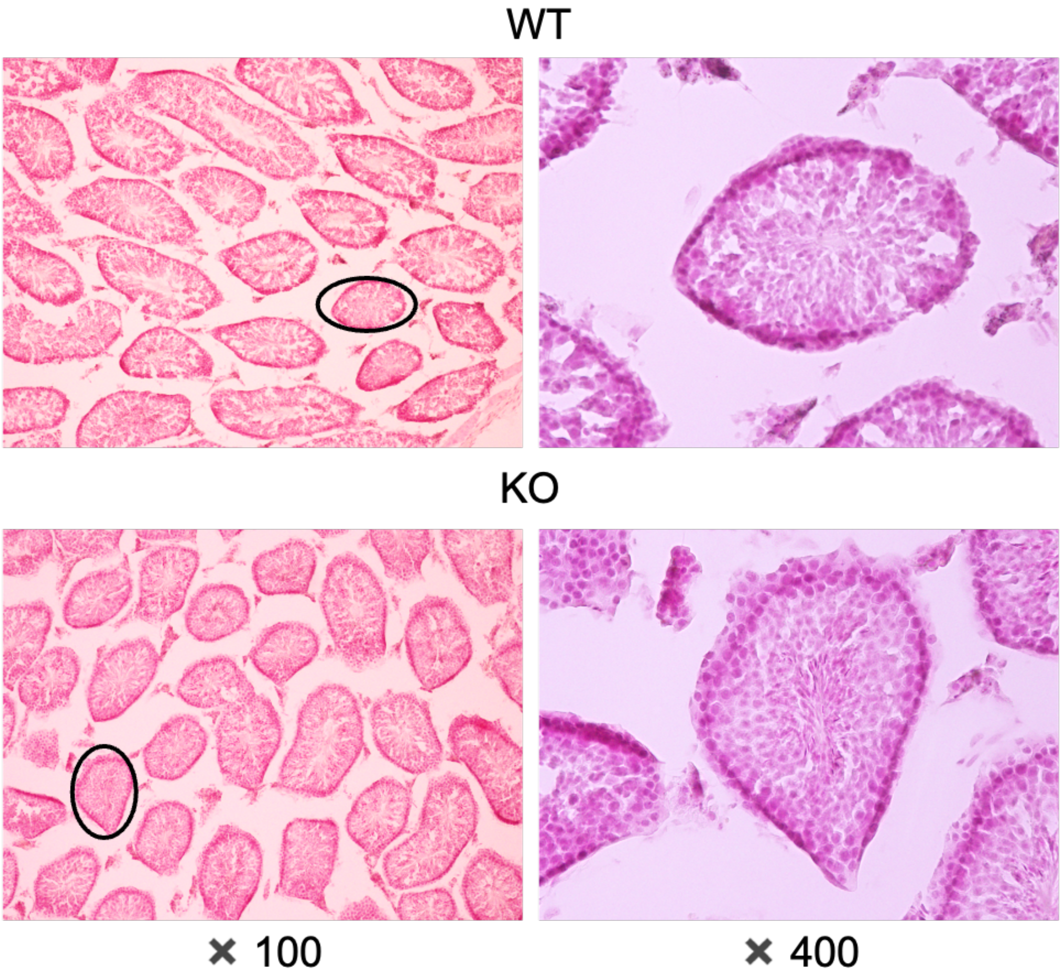

Fig. S4. Histopathological changes of mouse testis at PND49 by ablation of DAPL1. Testis sections (18 µm) were stained by hematoxylin and eosin solution

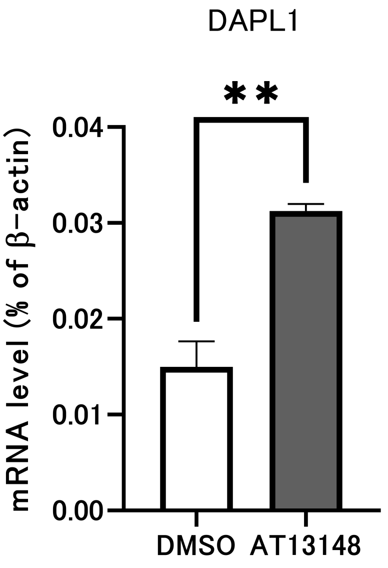

Fig. S5. Effect of 100 nM TA13148 on the gene-expression of mRNA coding for DAPL1 in I-10 cells. Each bar represents the mean ± S.E.M. of 4 samples. Significantly different from the DMSO group: **p < 0.01. DAPL1, death-associated protein-like 1; DMSO, dimethyl sulfoxide.

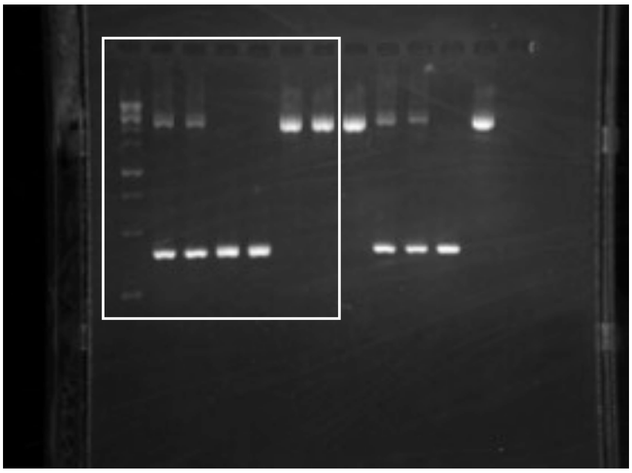

Fig. S6. Original blot of Figure 2B

Full length image of agarose gel: amplified DNAs were visualized under UV with ethidium bromide. Area of the gel image cropped for Figure 2B shown in rectangle.

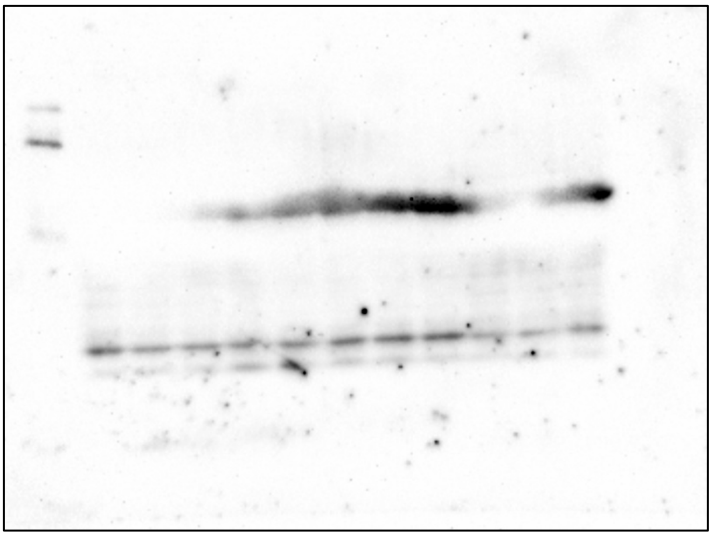

Fig. S7. Original blot of Figure 3B (StAR)

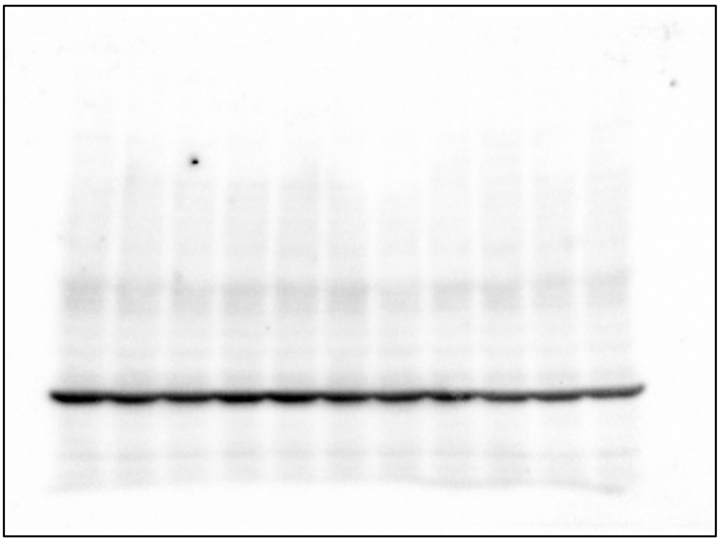

Fig. S8. Original blot of Figure 3B (β-actin)
